# Intermediate introns in nuclear genes of euglenids – are they a distinct type?

**DOI:** 10.1186/s12862-016-0620-5

**Published:** 2016-02-29

**Authors:** Rafał Milanowski, Natalia Gumińska, Anna Karnkowska, Takao Ishikawa, Bożena Zakryś

**Affiliations:** Department of Molecular Phylogenetics and Evolution, Institute of Botany, Faculty of Biology, University of Warsaw, Warsaw, Poland; Department of Botany, University of British Columbia, Vancouver, Canada; Department of Molecular Biology, Institute of Biochemistry, Faculty of Biology, University of Warsaw, Warsaw, Poland

**Keywords:** Euglenids, Nonconventional introns, Conventional spliceosomal introns, Intermediate introns, *tubB*, *hsp90*, *gapC*

## Abstract

**Background:**

Nuclear genes of euglenids contain two major types of introns: conventional spliceosomal and nonconventional introns. The latter are characterized by variable non-canonical borders, RNA secondary structure that brings intron ends together, and an unknown mechanism of removal. Some researchers also distinguish intermediate introns, which combine features of both types. They form a stable RNA secondary structure and are classified into two subtypes depending on whether they contain one (intermediate/nonconventional subtype) or both (conventional/intermediate subtype) canonical spliceosomal borders. However, it has been also postulated that most introns classified as intermediate could simply be special cases of conventional or nonconventional introns.

**Results:**

Sequences of *tubB*, *hsp90* and *gapC* genes from six strains of *Euglena agilis* were obtained. They contain four, six, and two or three introns, respectively (the third intron in the *gapC* gene is unique for just one strain). Conventional introns were present at three positions: two in the *tubB* gene (at one position conventional/intermediate introns were also found) and one in the *gapC* gene. Nonconventional introns are present at ten positions: two in the *tubB* gene (at one position intermediate/nonconventional introns were also found), six in *hsp90* (at four positions intermediate/nonconventional introns were also found), and two in the *gapC* gene.

**Conclusions:**

Sequence and RNA secondary structure analyses of nonconventional introns confirmed that their most strongly conserved elements are base pairing nucleotides at positions +4, +5 and +6/ -8, −7 and −6 (in most introns CAG/CTG nucleotides were observed). It was also confirmed that the presence of the 5' GT/C end in intermediate/nonconventional introns is not the result of kinship with conventional introns, but is due to evolutionary pressure to preserve the purine at the 5' end. However, an example of a nonconventional intron with GC-AG ends was shown, suggesting the possibility of intron type conversion between nonconventional and conventional. Furthermore, an analysis of conventional introns revealed that the ability to form a stable RNA secondary structure by some introns is probably not a result of their relationship with nonconventional introns. It was also shown that acquisition of new nonconventional introns is an ongoing process and can be observed at the level of a single species. In the recently acquired intron in the *gapC* gene an extended direct repeats at the intron-exon junctions are present, suggesting that double-strand break repair process could be the source of new nonconventional introns.

**Electronic supplementary material:**

The online version of this article (doi:10.1186/s12862-016-0620-5) contains supplementary material, which is available to authorized users.

## Background

Euglenids (Euglenida) are marine and freshwater unicellular flagellates with diverse modes of nutrition, including phagotrophy, osmotrophy and phototrophy. They are members of the Euglenozoa within the supergroup Excavata [1, 2]. Plastids of the phototrophic species, surrounded by three membranes, originated through secondary endosymbiosis between an eukaryovorous euglenid and a green alga closely related to members of the genus *Pyramimonas* [3–5]. Despite many years of study, knowledge about the organization of the genetic material in euglenids is still limited. While their chloroplast genomes have been well characterized [5–13], information on their nuclear genomes is rudimentary.

Nuclear genomes of all eukaryotes studied so far (with a few exceptions, e.g. [14, 15]) contain introns excised by the ribonucleoprotein complex, the spliceosome. Such conventional spliceosomal introns are also present in nuclear genes of euglenids. They have been found in the following genes: *nop1p* [16, 17], *tubA*, *tubB*, *tubG* [18–20], *hsp90* [21], *eno29*, *gapA*, *pbgD*, *petA*, *psaF*, *psbM*, and *psbW* [22]. However, conventional spliceosomal introns are not the only type of intervening elements in most of the analyzed genes of euglenids – they also contain another type of intron, termed nonconventional. Introns of this sort have non-canonical borders and form a stable RNA secondary structure bringing both ends together. The presence of nonconventional introns has been confirmed in such genes as *lhcp2* [23], *rbcS* [24], *gapC* [25], *tubA*, *tubB* [19, 20], *hsp90* [21], *petJ*, and *psbW* [22]. The length of nonconventional introns is variable, ranging from tens of bases to more than a kilobase; their RNA secondary structure is rather weakly preserved compared to other non-spliceosomal introns for which appropriate conformation is crucial for splice sites identification and proper removal (group I, II, and III self-splicing introns, tRNA introns, etc.). The most strongly conserved nucleotides are at positions +4, +5 and +6 at the 5' end of the intron and the complementary nucleotides −8, −7 and −6 at the 3' end. In addition, the following rules seem to apply to most of these introns: (i) a pyrimidine at the 3' end of the upstream exon and at the 3’ end of the intron, (ii) a purine at the 5' end of the intron and at the 5' end of the downstream exon, and (iii) cytosine at the third position of the downstream exon [20]. The mechanism of nonconventional intron removal is unknown, but the spliceosome appears to be uninvolved in excision due to a lack of complementarity between their 5' ends and U1 snRNA [16]. The origin of nonconventional introns is also a mystery, although based on the presence of inverted repeats near their ends and the frequent representation of direct repeats, insertion of MITE-like transposable elements has been proposed as a possible source [20]. An analysis of *tubA* and *tubB* genes from 20 species of euglenids has revealed contrasting distribution patterns of conventional and nonconventional introns. While the positions of conventional introns were conserved, those of nonconventional ones were unique to individual species or clades grouping closely related taxa [20].

In 2001 Canaday et al. distinguished a third type of nuclear intron in euglenids, so-called intermediate introns. They form a stable secondary structure bringing together intron ends and one or even both of their borders are consistent with the GT/C-AG rule characteristic for conventional introns [19]. It has been hypothesized that intermediate introns could be a transitional form between conventional and nonconventional introns [17] and could be recognized by two different mechanism of intron excision [20]. The subdivision of intermediate introns into two groups, conventional/intermediate and intermediate/nonconventional, was proposed based on an analysis of intron sequences and their distribution in tubulin genes. The former group includes cases with junctions typical for conventional introns and the potential to form a stable RNA secondary structure bringing intron ends together. The latter group includes introns with one conventional junction (usually at the 5' end) and a stable RNA secondary structure formed by intron ends consistent with the model of nonconventional intron junctions [20]. However, on the basis of previously published data it is not clear whether the features of intermediate introns reflect their kinship with both conventional and nonconventional introns or if they are the effects of other processes.

In order to shed new light on this issue, we analyzed intron sequences of *hsp90*, *tubB*, and *gapC* genes of six *Euglena agilis* strains. A comparison of homologous sequences from different strains of a species can help to identify the most conserved features of introns, thereby verifying intron provenance. *E. agilis* is a perfect choice for such an analysis due to its well documented high genetic variability [26–29].

## Results and discussion

### *tubB* gene

**Fig. 1:**
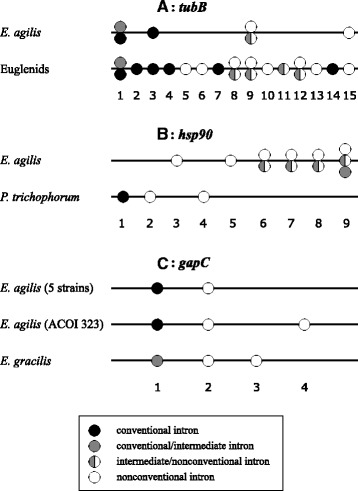
Intron positions in three genes of *E. agilis* and other euglenids. **a** *tubB*, **b** *hsp90*, **c** *gapC*. Circles indicate types of introns: black – conventional introns, gray – conventional/intermediate, gray|white – intermediate/nonconventional, white – nonconventional

Six *tubB* sequences from five *E. agilis* strains were obtained, encompassing 86 % of the coding region (two forms of the gene were obtained for the PO strain, differing by two point mutations within introns). The obtained sequences were compared with *tubB* sequences from the *E. agilis* ACOI 2970 reference strain. All the sequences contain four introns at the same positions (1, 3, 9 and 15 according to the numbering of introns in the *tubB* gene from euglenids, Fig. 1a; [20]). (Additional file 1: Table S1) shows sequences of all intron-exon junctions in the *tubB* gene.

In all the species studied so far, the intron at position 1 has GT-AG boundaries and the ability to form a stable secondary structure has not been detected with the exception of the *E. agilis* ACOI 2970 strain [20]. In the sequences obtained in this study, the GT-CAG boundaries of intron 1 are preserved and a stable RNA secondary structure bringing together intron ends can be formed only by the intron from the PO strain. The intron at position 3 occurs only in those species in which intron 1 is also present [20]. In all the cases examined earlier as well as in the *E. agilis* strains studied herein, intron 3 has GT-AG ends and cannot form a stable RNA secondary structure bringing its ends together. Thus, position 3 contains a typical conventional spliceosomal intron. The intron located at position 9 is present only in closely related species *Euglena gracilis*, *Euglena longa*, and *E. agilis*. In *E. longa* it is a typical nonconventional intron, whereas in *E. gracilis* and *E. agilis* strain ACOI 2970 it has been classified as intermediate/nonconventional because of the GT 5' end [20]. In the strains studied herein, a stable RNA secondary structure of introns at position 9 and all elements characteristic for nonconventional intron were detected (Additional file 1: Table S1). Additionally, in comparison to introns at positions 1 and 3, much greater length variation was observed (1: 49–57, 3: 44–50, 9: 52–542 bp). Furthermore, at the 5' end of the intron a purine (G or A) is always present, while the following nucleotide is not conserved; a GT end is found in ACOI 2970 and WA strains whereas a GC end is present in the WI strain. A purine is located at the 3' end of the introns of PR and WI strains and this is the only deviation from the model of nonconventional intron junctions. The intron at position 15 is observed only in *tubB* genes from four species of the *Euglena* genus: *Euglena gymnodinioides*, *E. gracilis*, *E. longa* and *E. agilis* [20]. In all these species it is a typical nonconventional intron lacking the GT/C-AG ends and able to form a stable RNA secondary structure consistent with the proposed model. This is also true for the *E. agilis* strains studied here. The only deviation from the model is nucleotide T (not C as in most cases) at the third position of the downstream exon in the gene from the PO strain (Additional file 1: Table S1). Similarly to intron 9, the variability of the length of this intron (15: 82–305) is much higher than for introns 1 and 3.

### *hsp90* gene

Amplification of the *hsp90* gene fragment encompassing 98 % of the coding region (using primers EA9F0/R0, EA9F1/R1; Additional file 1: Table S4) was successful only for the PO strain (4758 bp). The obtained sequence contains six introns. New primers were designed encompassing a shorter gene fragment with all the introns defined for the PO strain (primers EA9s F0/R0/F1/R1; Additional file 1: Table S4). Amplification with EA9s F0/R0 and EAsF1/R1 primers was successful for ACOI 323 (4027 bp), PR (4133 bp), WI (3308 bp) and WA (2721 bp) strains. The gene fragment from the ACOI 2970 strain was amplified in two parts using EA9sF0/R2, EA9sF1/R3 and EA9sF2/R0, EA9sF3/R1 primers (assembled sequence: 7977 bp). All of the sequences contain six introns at the same positions. However, all introns from *E. agilis* are at different positions than the three introns in the *Peranema trichophorum* (primary heterotrophic species) gene, the only known *hsp90* gene from an euglenid (one conventional and two nonconventional introns; Fig. 1b). Table S2 (Additional file 1) shows sequences of intron-exon junctions in the *hsp90* gene.

The stable RNA secondary structure characteristic for nonconventional intron ends can be formed by all the introns in the *hsp90* gene from all the examined *E. agilis* strains. Additionally, the presence of a purine at the 5' end of downstream exons was observed in all introns. A few deviations were observed for other attributes of nonconventional intron junctions in the analyzed sequences: (i) a purine at the 3' end of the upstream exon of intron 6 from all strains (numbering of introns in the *hsp90* gene according to the scheme in Fig. 1b), (ii) a pyrimidine at the 5' end of intron 3 from ACOI 323, PR, PO and WA strains, (iii) a purine at the 3' end of introns 5 and 6 from ACOI 2970, intron 7 from WI and intron 9 from ACOI 323, (iv) a non-cytosine nucleotide at the third position of the downstream exon of introns 5, 6, 7 and 9 from all strains. The GC 5' end was found in 10 introns (intron 6 from ACOI 2970, ACOI 323, PR, PO, WA; intron 7 from PR; introns 8 and 9 from ACOI 323 and WA). Moreover, intron 9 from the ACOI 323 strain has GC-AG ends characteristic for the conventional type. High variation in intron length was observed in introns at positions 5, 6, 7, 8, and 9 (3: 44–50, 5: 125–1148, 6: 153–1077, 7: 140–384, 8: 438–1953, 9: 247–2343 bp).

### *gapC* gene

Nine *gapC* sequences from six *E. agilis* strains were obtained, encompassing the coding region for which three introns were defined in the gene from *E. gracilis* (two forms of the gene, slightly different, were obtained for strains ACOI 323, WI, and PO). Sequences from five strains of *E. agilis* contain two introns at the same positions as in the *E. gracilis* gene. Sequences from the ACOI 323 strain contain a third additional intron at a new position. Table S3 (Additional file 1) shows sequences of all intron-exon junctions in the *gapC* genes from *E. agilis*.

The intron at position 1 in the sequence of the *gapC* gene from *E. gracilis* (numbering of introns in the *gapC* gene according to the scheme in Fig. 1c) was described as nonconventional because of its ability to form a stable secondary structure and despite GT-AG ends. At the moment of publication only nonconventional introns were known in genes of euglenids [25]. However, the secondary structure of this intron resembles – to a small extent – the structure of nonconventional introns, therefore it should be classified as a conventional/intermediate rather than simply a nonconventional one. All *gapC* gene sequences obtained in this study contain introns at position 1; all of them have GT-AG ends and the ability to form a stable secondary structure was not detected. Thus, they should be classified as conventional introns. All the examined introns at position 2 can form a stable secondary structure bringing intron ends together and do not obey the GT/C-AG rule; also other elements characteristic for nonconventional introns are present (Additional file 1: Table S3).

Analysis of intron distribution in *tubA* and *tubB* genes revealed that nonconventional introns appear in nuclear genes of euglenids quite often and many of them are unique for single species [20]. Results presented herein allow for an even more far-reaching conclusion – nonconventional intron gain is an ongoing process that can be observed at the level of a single species. The *gapC* gene of strain ACOI 323 contains an intron at position 4 which is absent in the sequences of other *E. agilis* strains, suggesting that this intron is a new structure and was acquired relatively recently. Intron 4 has all of the features of nonconventional introns. Interestingly, eight nucleotide direct repeats were observed at the intron-exon junction (longer than in the case of other introns analyzed herein; Additional file 1: Table S3). Similarly, short direct repeats are also present at the intron-exon junctions of recently acquired conventional introns in several genes from *Daphnia pulex*, explained as the fingerprint of intron gain by the double-strand break repair process [30]. It is worth considering if this process could also be the source of new nonconventional introns. This is particularly interesting in the case of euglenids because of their remarkable resistance to UV and ionizing radiation related to high double-strand break rejoining activity [31].

### Identification of nonconventional introns

The recognition of the borders of nonconventional intron is not trivial due to the lack of close end preservation, even when compared to the sequence of the mature transcript. Direct repeats often observed at intron-exon junctions make intron position difficult to determine precisely. This is why the boundaries of many nonconventional introns deposited in databases have not been defined correctly. A comparison of all known intron sequences and elucidation of the common characteristics of non-conventional introns greatly improved our understanding [20], although in many introns, including those studied in this work, numerous deviations from the common model are observed.

**Fig. 2:**
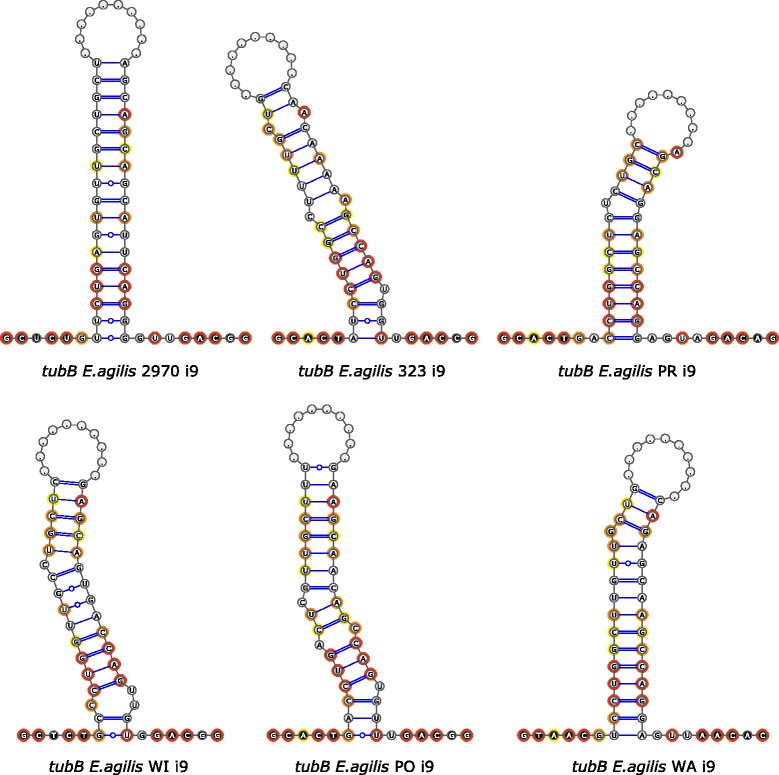
Homologous introns at position 9 in *tubB* genes. A comparison of RNA secondary structure of introns at position 9 in *tubB* genes from six strains of *E. agilis*. The middle part of the sequence is omitted and indicated by dots. Exons in black circles, introns in gray; nucleotides present in all six sequences highlighted in red, in five of six in orange, in four of six in yellow. Classical Watson-Crick base-pairing interactions are marked by single (AU) or double (GC) lines, non-canonical GU pairs by single line with circle. Note: the U/A base pair is present at positions +5/-7 instead of A/U observed in other nonconventional introns

**Fig. 3:**
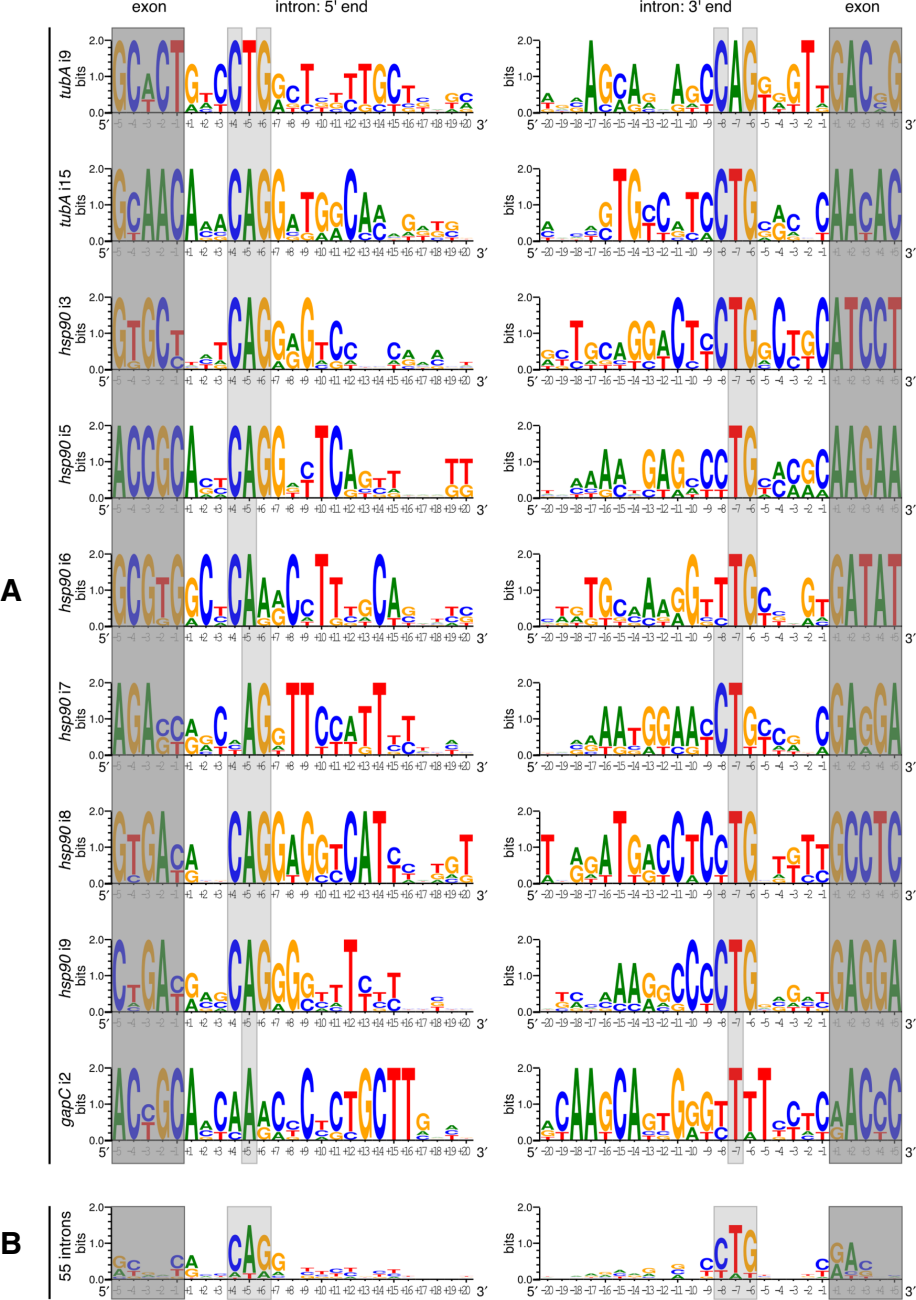
Sequence logos. **a** Sequence logos of exon/intron junctions of nine homologous nonconventional introns. **b** Sequence logo of exon/intron junctions of all 55 analyzed nonconventional introns; introns in different forms of the gene from the same strain not included. Exons shaded in dark gray; conserved nucleotides CAG/CTG at positions +4, 5, 6/-8, 7, 6 shaded in light gray

The ends of all nonconventional introns studied herein, as well as all others known so far, form a stable secondary structure bringing together intron ends and the ends of adjacent exons. Nevertheless, comparisons of homologous introns indicate that this structure is conserved to a very limited extent. The ends of some introns form strict stems of several nucleotides without any internal loops or bulges (for example, intron 9 in the *tubB* gene from strain ACOI 2970, intron 3 in *hsp90* from WI and WA), while in homologous sequences unpaired nucleotides at different positions are observed (Fig. 2). The presence of many compensatory base changes between homologous sequences underlines the significance of secondary structure, although mutations creating internal loops or bulges were also found. The only nucleotides which always form base pairs in the analyzed nonconventional introns are present at positions +5, +6 and −8, −7 (55 sequences; homologous introns in different forms of the gene from the same strain are not included); nucleotides at positions +4 and −6 are unpaired only in one sequence. In most introns CAG/CTG nucleotides are found at these positions (33 of 55 sequences), in agreement with a previous study for all known nonconventional introns [20] (Fig. 3). Other stem-forming nucleotides are also conserved in several cases, but only within the groups of homologous introns, not between different introns (with the exception of positions +7/-9 where G/C is observed in 31 of 55 sequences). This means that close location of junctions, the effect of intron end base pairing, is crucial for splicing site recognition, although no distinct common motif in intron secondary structure is noticeable. The question is whether CAG(G)/(C)CTG nucleotides observed in most but not all nonconventional introns act as a signal of key importance in the process of intron excision or if they are just the “fingerprint” of the source sequence of all nonconventional introns. In the latter case, these nucleotides could be changed in time by compensatory mutations appearing at a slower rate due to the importance of this process for maintenance of proper secondary structure.

The base pairing of ends is not the only feature of nonconventional introns, i.e. nucleotides at the close ends of exons and introns are also conserved to a certain extent: purines at the 5' end of an intron (51 of 55 analyzed introns) and at the 5' end of a downstream exon (all sequences) and pyrimidines at the 3' end of an upstream exon (49 of 55) and 3' of an intron (49 of 55). This pattern suggests that deviations from the model at the end positions of exons and introns are tolerated. Alterations at the third position of the downstream exon were also observed – C is present only in 30 of 55 analyzed sequences. Notably, an A at the second position of downstream exons is more conserved than the C at the third in 43 of 55 sequences (Fig. 3). It seems that nucleotide identity at the exon and intron ends are rather weak splicing determinants that can be recognized only when the secondary structure of intron ends is formed. The question whether slightly conserved *cis* signals are sufficient for defining exon-intron borders remains open. Participation of *trans* factors, such as antisense RNA molecules, should also be taken into account.

#### Intermediate introns – true or false?

**Fig. 4:**
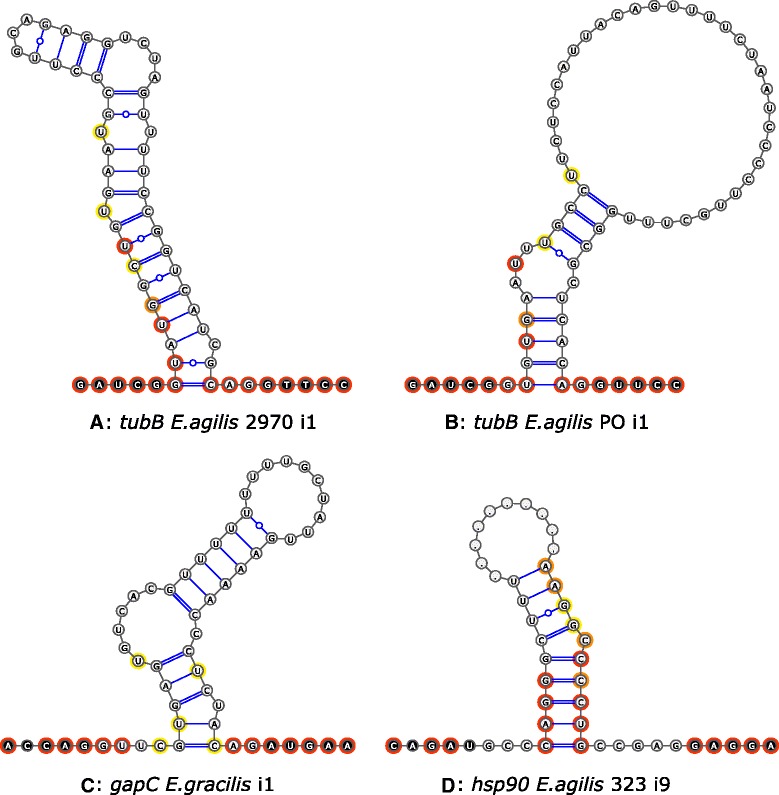
RNA secondary structure of introns. **a** and **b** Introns at position 1 in *tubB* genes from *E. agilis* (strains ACOI 2970 and PO, respectively). **c** Intron at position 1 in *gapC* gene from *E. gracilis* (secondary structure according to [25]). **d** intron at position 9 in the *hsp90* gene from *E. agilis* strain ACOI 323 (most nucleotides from the middle part omitted and indicated by dots). Exons in black circles, introns in gray; nucleotides present in all six homologous sequences of *E. agilis* highlighted in red, in five of six in orange, in four of six in yellow. Classical Watson-Crick hydrogen bonding marked by single (AU) or double (GC) lines, non-classical GU pairs by single line with circle

Intermediate introns have characteristics of both conventional and nonconventional types. Introns at position 1 in *tubB* genes from *E. agilis* ACOI 2970 and PO strains were classified as conventional/intermediate with both borders typical for conventional introns and the potential to form a stable RNA secondary structure. A comparison of the modeled RNA secondary structures of these introns shows no common elements; characteristic features of nonconventional introns are not apparent either (Fig. 4a and b). GT-CAG ends as well as a few other nucleotides near the 5' end are essentially the only conserved features of introns at this position from all strains. As mentioned earlier, intron 1 in the *gapC* gene of *E. gracilis* should also be classified as conventional/intermediate. However, the only features in common with nonconventional introns are purines at the 5' ends of the intron and the downstream exon (Fig. 4c). Homologous introns 1 from *E. agilis* strains obtained in this study are typical conventional types and the secondary structure required to bring together intron ends was not detected in any of them. This indicates that the introns at position 1 in *gapC* and in *tubB* genes are conventional spliceosomal introns. Moreover, the ability to form a stable RNA secondary structure by some is probably not a result of their relationship with nonconventional introns. It is assumed that the secondary structure sometimes observed in conventional introns of many organisms could facilitate their splicing [32–34], although its presence in conventional introns of euglenids could also have a different explanation. Previous studies have suggested that nonconventional introns are excised before conventional ones [24]. The presence of conventional and nonconventional introns in the same gene could disturb the process of nonconventional intron folding. For instance, the ability of conventional introns to form a secondary structure could facilitate the correct folding of nonconventional introns and their fast excision. However, the primary determinant of the assignment of intron to type should be the mechanism of intron excision. It cannot be excluded that conventional/intermediate introns, such as introns 1 in *tubB* and *gapC* genes, could be excised from the primary transcript by both spliceosomal and nonconventional mechanisms, in the latter case on lower level. It cannot be excluded also that other nuclear genes of euglenids contain conventional/intermediate introns forming secondary structure consistent with the slightly conserved model for nonconventional introns. Such hypothetical intron would be the example of the real intermediate intron excised by two mechanisms.

Thirteen of fifty five analyzed introns meeting the characteristics of the nonconventional type have GT or GC 5' ends, meaning that they should be classified as intermediate/nonconventional introns. It has been hypothesized that the presence of GT or GC ends may not be the result of the relationship with conventional introns, but rather an effect of evolutionary pressure to preserve the purine at the 5' end (then followed by any nucleotide), a feature of most nonconventional introns [20]. An analysis of 55 introns in *tubB*, *hsp90*, and *gapC* genes confirms this hypothesis. Most nonconventional introns (51 of 55 analyzed) have a purine at the +1 position, while nucleotides at positions +2 and +3 are not conserved between different introns, as well as between homologous introns from closely related taxa (with a few minor exceptions; Fig. 3). The presence of the GT/C end is not in itself evidence of kinship to conventional introns. Such sequences should rather be considered as nonconventional introns susceptible to potential transformation into conventional ones. Is this transformation possible? All analyzed introns at position 9 in *hsp90* have typical features of nonconventional introns with one exception, i.e. the intron from ACOI 323 strain has an unusual AG 3' end, while the 5' end of the intron is GC (Fig. 4d). Thus, this intron may be removed by two mechanisms. If this is confirmed, then this intron should be defined as intermediate. Notwithstanding the foregoing discussion, intron 9 from the ACOI 323 strain of *E. agilis* is the first example of a nonconventional intron which potentially could also be excised by the spliceosome with the same cleavage sites. This also means that the change of intron type from nonconventional to conventional is possible.

## Conclusions

In this paper we analyzed thirteen introns in *tubB*, *hsp90*, and *gapC* genes from six strains of *E. agilis*. Sequence and RNA secondary structure analyses of the nonconventional introns confirmed that the most strongly conserved elements are base pairing nucleotides at positions +4, +5 and +6/ -8, −7, and −6. Their complementarity is maintained rather than sequence conservation, although in most introns CAG/CTG nucleotides were observed; the close ends of these introns are preserved to a lesser extent.

It was shown that the presence of the 5' GT/C end of intermediate/nonconventional introns is not the result of their kinship with conventional introns, but emerges as a result of the evolutionary pressure to preserve the purine at their end (followed by any nucleotide), a characteristic for most nonconventional introns. An intermediate intron candidate containing features of both types of introns was also discussed.

The presence of recently acquired nonconventional introns in the *gapC* gene from one strain of *E. agilis* indicates that nonconventional intron acquisition is an ongoing process and can be observed at the level of a single species. In the recently acquired intron in the *gapC* gene an extended direct repeats at the intron-exon junctions are present, what suggests that double-strand break repair process could be the source of new nonconventional introns.

## Methods

### Strains and culture conditions

Frozen cells of six *E. agilis* clones were used for this study: two clones were derived from Portugal strains (ACOI 323 and ACOI 2970, Culture Collection of Algae at the Department of Botany, University of Coimbra, Portugal) and four from natural populations collected throughout Poland in 1992–1993, for which morphological and genetic analyses (RAPD and RFLP) have been performed [26, 27]. The Polish strains (clones) were named according to the localities from which they were collected: PO (puddle in village Połom, northeastern Poland near Ełk; 53° 58' 31,3" N, 22° 16' 19,6" E), PR (sewer canal in town Pruszków near Warsaw; 52° 09' 57,4" N, 20° 49' 0,3" E), WA (pond in village Wąwocko, southern Poland; 51° 17' 55,1" N, 22° 08' 28,4" E) and WI (Wisła River, beach, 3 km north of Warsaw; 52°19' 22,9" N, 20° 55' 45,7" E); for additional information see Fig. 1. and Table 1. in [27].

### Molecular techniques

DNA extraction and PCR amplification of *tubB* were performed as described previously [20]. Nested PCR amplifications of the *hsp90* gene were done with primers based on cDNA sequences from *E. agilis* ACOI 2970 [GenBank: KT984750-1]. Sequences of all the primers used in this study are listed in (Additional file 1: Table S4). Four sets of primers were used: (1) EA9F0/R0 (external pair, annealing temperature 68 **°**C) and EA9F1/R1 (internal, 58 **°**C) covering almost the entire gene; (2) EA9sF0/R0 (external, 60 **°**C) and EA9sF1/R1 (internal, 59 **°**C) covering the fragment of gene with introns; (3) EA9sF0/R2 (external, 55 **°**C) and EA9sF1/R3 (internal, 55 **°**C) covering the first part of the gene; (4) EA9sF2/R0 (external, 55 **°**C) and EA9sF3/R1 (internal, 55 **°**C) covering the second part of the gene. Based on the cDNA sequence of the *gapC* gene from *E. agilis* ACOI 2970 [GenBank: KT962995], two pairs of primers were designed: EAgCF0/R0 (external, 62 **°**C) and EAgCF1/R1 (internal, 60 **°**C) which cover the fragment of the gene where three introns were identified previously in the sequence of *E. gracilis*. Conditions of nested PCR: in the first PCR reaction, a 25 μl reaction mixture contained 0.5 U Phusion High-Fidelity DNA Polymerase (Thermo Scientific), 0.2 mM dNTPs, 3.5 mM MgCl_**2**_, 10 pmol of each primer, reaction buffer GC (Thermo Scientific), Q-solution (Qiagen), 1.2 μg Taq Single-Stranded DNA Binding Protein (EURx) and 10–50 ng DNA. The PCR protocol consisted of 2 min at 98 °C, followed by seven initial cycles comprising 30 s at 98 **°**C, 30 s at 55–68 **°**C (depending on the set of primers) and 2.5 min at 72 **°**C, then by 35 cycles comprising 15 s at 98 **°**C, 15 s at 55–68 **°**C and 2.5 min at 72 **°**C. The final extension step was performed for 5 min at 72 **°**C. In the second PCR reaction, a 25 μl reaction mixture contained 0.5 U Phusion High-Fidelity DNA Polymerase (Thermo Scientific), 0.2 mM dNTPs, 1.5 mM MgCl_**2**_, 10 pmol of each primer, reaction buffer GC (Thermo Scientific), Q-solution (Qiagen), 0.6 μg Taq Single-Stranded DNA Binding Protein (EURx) and 1 μl of undiluted mixture from the first step. The PCR protocol consisted of an initial 2 min at 98 °C, followed by 35 cycles comprising 15 s at 98 °C, 15 s at 55–60 °C and 2.5 min at 72 °C. PCR products were cloned in a pGem-T Easy vector and sequenced as described previously [20]. M13F and M13R universal primers were used for sequencing, as well as internal primers corresponding to the regions in the middle of the genes (see Additional file 1: Table S4).

### Sequence analysis

The sequence readings were assembled into contigs by the SeqMan program (Lasergene package, DnaStar). To determine intron positions, a comparison of genomic and cDNA sequences was made in Mesquite [35]. The alignments of both intron ends for logo analysis (20 nucleotides each) were prepared manually without any indels. Prediction of the RNA secondary structure of intron ends was done using the RNAfold WebServer [36] with default options; obtained structures were corrected manually according to the structure of homologous sequences if needed (a few cases). The RNA secondary structures were drawn using Varna 3.9 [37]. Nucleotide logos of intron junctions were created using Weblogo 3 [38].

## Availability of data and materials

The data set supporting the results of this article is included within the article and its Additional file 1. GenBank accession numbers for the sequences reported in this paper: *tubB* [GenBank: KM262225-30], *hsp90* [GenBank: KU041762-7], *gapC* [GenBank: KU041768-76]. GenBank accession numbers for the *tubB* sequences from *E. agilis* strain ACOI 2970 are [GenBank: KC907661-3].

## Additional file

Additional file 1:
**Table S1.** Exon-intron junctions in *tubB* genes from six strains of *E. agilis*. Abbreviations: pos – position of intron; name – abbreviation of taxon name (if more than one form of gene was found, number is added); type – type of intron (C – Conventional, C/I – Conventional/Intermediate, I/N – Intermediate/Nonconventional, N – Nonconventional); size –length of intron in bp; sequence – partial sequence of exon-intron junction. Exon sequences in upper case, intron in lower case; direct repeats shaded; if present, GT/C-AG ends of introns in bold; nucleotides involved in forming the intron secondary structure underlined. **Table S2.** Exon-intron junctions in *hsp90* genes from six strains of *E. agilis*. For description see Table S1. **Table S3.** Exon-intron junctions in *gapC* genes from six strains of *E. agilis*. For description see table S1. **Table S4.** Primers used for amplification and sequencing of *hsp90* and *gapC* genes. (PDF 120 kb)

## References

[1] Hampl V, Hug L, Leigh JW, Dacks JB, Lang BF, Simpson AGB, Roger AJ. Phylogenomic analyses support the monophyly of Excavata and resolve relationships among eukaryotic “supergroups”. Proc Natl Acad Sci USA. 2009;106:3859–64. 10.1073/pnas.0807880106 PMC2656170 19237557

[2] Adl SM, Simpson AGB, Lane CE, Lukeš J, Bass D, Bowser SS, Brown MW, Burki F, Dunthorn M, Hampl V, Heiss A, Hoppenrath M, Lara E, Le Gall L, Lynn DH, McManus H, Mitchell E a D, Mozley-Stanridge SE, Parfrey LW, Pawlowski J, Rueckert S, Shadwick L, Schoch CL, Smirnov A, Spiegel FW. The revised classification of eukaryotes. J Eukaryot Microbiol. 2012;59:429–93. 10.1111/j.1550-7408.2012.00644.x PMC3483872 23020233

[3] Turmel M, Gagnon M-C, O’Kelly CJ, Otis C, Lemieux C (2009). The chloroplast genomes of the green algae *Pyramimonas*, *Monomastix*, and *Pycnococcus* shed new light on the evolutionary history of prasinophytes and the origin of the secondary chloroplasts of euglenids. Mol Biol Evol.

[4] Yamaguchi A, Yubuki N, Leander BS (2012). Morphostasis in a novel eukaryote illuminates the evolutionary transition from phagotrophy to phototrophy: description of *Rapaza viridis* n. gen. et sp. (Euglenozoa, Euglenida). BMC Evol Biol.

[5] Hrdá Š, Fousek J, Szabová J, Hampl V, Vlček Č (2012). The plastid genome of *Eutreptiella* provides a window into the process of secondary endosymbiosis of plastid in euglenids. PLoS ONE.

[6] Hallick RB, Hong L, Drager RG, Favreau MR, Monfort A, Orsat B, Spielmann A, Stutz E. Complete sequence of *Euglena gracilis* chloroplast DNA. Nucleic Acids Res. 1993;21:3537–44. 10.1093/nar/21.15.3537 PMC331456 8346031

[7] Gockel G, Hachtel W (2000). Complete gene map of the plastid genome of the nonphotosynthetic euglenoid flagellate *Astasia longa*. Protist.

[8] Wiegert KE, Bennett MS, Triemer RE (2012). Evolution of the chloroplast genome in photosynthetic euglenoids: a comparison of *Eutreptia viridis* and *Euglena gracilis* (Euglenophyta). Protist.

[9] Pombert J-F, James ER, Janouškovec J, Keeling PJ (2012). Evidence for transitional stages in the evolution of euglenid group II introns and twintrons in the *Monomorphina aenigmatica* plastid genome. PLoS ONE.

[10] Bennett MS, Wiegert KE, Triemer RE (2012). Comparative chloroplast genomics between *Euglena viridis* and *Euglena gracilis* (Euglenophyta). Phycologia.

[11] Wiegert KE, Bennett MS, Triemer RE (2013). Tracing patterns of chloroplast evolution in euglenoids: contributions from *Colacium vesiculosum* and *Strombomonas acuminata* (Euglenophyta). J Eukaryot Microbiol.

[12] Bennett M, Wiegert K, Triemer R (2014). Characterization of *Euglenaformis* gen. nov. and the chloroplast genome of *Euglenaformis proxima* (Euglenophyta). Phycologia.

[13] Bennett MS, Triemer RE (2015). Chloroplast genome evolution in the Euglenaceae. J Eukaryot Microbiol.

[14] Lane CE, van den Heuvel K, Kozera C, Curtis B a, Parsons BJ, Bowman S, Archibald JM. Nucleomorph genome of *Hemiselmis andersenii* reveals complete intron loss and compaction as a driver of protein structure and function. Proc Natl Acad Sci USA. 2007;104:19908–13. 10.1073/pnas.0707419104 PMC2148396 18077423

[15] Keeling PJ, Corradi N, Morrison HG, Haag KL, Ebert D, Weiss LM, Akiyoshi DE, Tzipori S. The reduced genome of the parasitic microsporidian *Enterocytozoon bieneusi* lacks genes for core carbon metabolism. Genome Biol Evol. 2010;2:304–9. 10.1093/gbe/evq022 PMC2942035 20624735

[16] Breckenridge DG, Watanabe Y-I, Greenwood SJ, Gray MW, Schnare MN (1999). U1 small nuclear RNA and spliceosomal introns in *Euglena gracilis*. Proc Natl Acad Sci U S A.

[17] Russell AG, Watanabe Y, Charette JM, Gray MW (2005). Unusual features of fibrillarin cDNA and gene structure in *Euglena gracilis*: evolutionary conservation of core proteins and structural predictions for methylation-guide box C/D snoRNPs throughout the domain Eucarya. Nucleic Acids Res.

[18] Ebel C, Frantz C, Paulus F, Imbault P (1999). Trans-splicing and cis-splicing in the colourless Euglenoid, *Entosiphon sulcatum*. Curr Genet.

[19] Canaday J, Tessier LH, Imbault P, Paulus F (2001). Analysis of *Euglena gracilis* alpha-, beta- and gamma-tubulin genes: introns and pre-mRNA maturation. Mol Genet Genomics.

[20] Milanowski R, Karnkowska A, Ishikawa T, Zakryś B (2014). Distribution of conventional and nonconventional introns in tubulin (α and β) genes of euglenids. Mol Biol Evol.

[21] Breglia SA, Slamovits CH, Leander BS (2007). Phylogeny of phagotrophic euglenids (Euglenozoa) as inferred from *hsp90* gene sequences. J Eukaryot Microbiol.

[22] Vesteg M, Vacula R, Steiner JM, Mateásiková B, Löffelhardt W, Brejová B (2010). A possible role for short introns in the acquisition of stroma-targeting peptides in the flagellate *Euglena gracilis*. DNA Res.

[23] Muchhal US, Schwartzbach SD (1994). Characterization of the unique intron - exon junctions of *Euglena* gene(s) encoding the polyprotein precursor to the light-harvesting chlorophyll a/b binding protein of photosystem II. Nucleic Acids Res.

[24] Tessier LH, Paulus F, Keller M, Vial C, Imbault P (1995). Structure and expression of *Euglena gracilis* nuclear *rbcS* genes encoding the small subunits of the ribulose 1,5-bisphoshate carboxylase/oxygenase: A novel splicing process for unusual intervening sequences?. J Mol Biol.

[25] Henze K, Badr A, Wettern M, Cerff R, Martin W (1995). A nuclear gene of eubacterial origin in *Euglena gracilis* reflects cryptic endosymbioses during protist evolution. Proc Natl Acad Sci USA.

[26] Zakryś B, Kucharski R (1996). Microevolutionary processes in *Euglena pisciformis*. Genetic drift or adaptation?. Arch Hydrobiol Suppl Algol Stud.

[27] Zakryś B, Kucharski R, Moraczewski I (1996). Genetic and morphological variability among clones of *Euglena pisciformis* based on RAPD and biometric analysis. Arch Hydrobiol Suppl Algol Stud.

[28] Zakryś B, Empel J, Milanowski R, Gromadka R, Borsuk P, Kędzior M, Kwiatowski J. Genetic variability of *Euglena agilis* (Euglenophyceae). Acta Soc Bot Pol. 2004;73:305–9.

[29] Milanowski R, Kosmala S, Zakryś B, Kwiatowski J (2006). Phylogeny of photosynthetic euglenophytes based on combined chloroplast and cytoplasmic SSU rDNA sequence analysis. J Phycol.

[30] Li W, Tucker AE, Sung W, Thomas WK, Lynch M (2009). Extensive, recent intron gains in *Daphnia* populations. Science.

[31] Hayashi H, Narumi I, Wada S, Kikuchi M, Furuta M, Uehara K, Watanabe H. Light dependency of resistance to ionizing radiation in *Euglena gracilis*. J Plant Physiol. 2004;161:1101–6. 10.1016/j.jplph.2004.04.005 15535119

[32] Chen Y, Stephan W (2003). Compensatory evolution of a precursor messenger RNA secondary structure in the *Drosophila melanogaster* Adh gene. Proc Natl Acad Sci USA.

[33] van der Burgt A, Severing E, de Wit PJGM, Collemare J (2012). Birth of new spliceosomal introns in fungi by multiplication of introner-like elements. Curr Biol.

[34] Roy SW, Hudson AJ, Joseph J, Yee J, Russell AG (2012). Numerous fragmented spliceosomal introns, AT-AC splicing, and an unusual dynein gene expression pathway in *Giardia lamblia*. Mol Biol Evol.

[35] Maddison WP, Maddison DR. Mesquite: a modular system for evolutionary analysis. Version 3.04. 2015. http://mesquiteproject.org. Accessed 12 Nov 2015.

[36] RNAfold Web Server. 2015. http://rna.tbi.univie.ac.at/cgi-bin/RNAfold.cgi. Accessed 12 Nov 2015.

[37] Darty K, Denise A, Ponty Y (2009). VARNA: Interactive drawing and editing of the RNA secondary structure. Bioinformatics.

[38] Crooks G, Hon G, Chandonia JM, Brenner SE (2004). WebLogo: a sequence logo generator. Genome Res.

