# Supplementary material for: Intermediate introns in nuclear genes of euglenids – are they a distinct type?
Source: BMC Evol Biol. 2016 Feb 29;16:49. doi: 10.1186/s12862-016-0620-5 (PMC4770533; doi:10.1186/s12862-016-0620-5)
Supplement: Additional file 1: — Table S1. Exon-intron junctions in tubB genes from six strains of E. agilis. Abbreviations: pos – position of intron; name – abbreviation of taxon name (if more than one form of gene was found, number is added); type – type of intron (C – Conventional, C/I – Conventional/Intermediate, I/N – Intermediate/Nonconventional, N – Nonconventional); size –length of intron in bp; sequence – partial sequence of exon-intron junction. Exon sequences in upper case, intron in lower case; direct repeats shaded; if present, GT/C-AG ends of introns in bold; nucleotides involved in forming the intron secondary structure underlined. Table S2. Exon-intron junctions in hsp90 genes from six strains of E. agilis. For description see Table S1. Table S3. Exon-intron junctions in gapC genes from six strains of E. agilis. For description see table S1. Table S4. Primers used for amplification and sequencing of hsp90 and gapC genes. (PDF 120 kb) [file 12862_2016_620_MOESM1_ESM.pdf]

Supplementary Table S1. Exon-intron junctions in *tubB* genes from six strains of *E. agilis*. Abbreviations: pos – position of intron; name – abbreviation of taxon name (if more than one form of gene was found, number is added); type – type of intron (C – Conventional, C/I – Conventional/Intermediate, I/N – Intermediate/Nonconventional, N – Nonconventional); size –length of intron in bp; sequence – partial sequence of exon-intron junction. Exon sequences in upper case, intron in lower case; direct repeats shaded; if present, GT/C-AG ends of introns in bold; nucleotides involved in forming the intron secondary structure underlined.

tubB

pos	name	type	size	sequence
1	agil_2970_1	C/I?	49	GATCG|**gt**atggctgtgaatgccctt...tagtttccggtcatcgc**ag**|GTTCC
1	agil_2970_2/3	C/I?	49	GATCG|**gt**atggctgtgaatgcccttt...taggtttccggtcatcgc**ag**|GTTCC
1	agil_323	C	52	GATCG|**gt**gtggctattatttgccc...ttgccgacatacgcatgc**ag**|GTTCC
1	agil_PR	C	52	GATCG|**gt**atgtcttttgaagcctttt...tcttcccctcaacgcatc**ag**|GTTCC
1	agil_WI	C	52	GATCG|**gt**atatctacatcttgcatt...ttcctcatcgtgccgctc**ag**|GTTCC
1	agil_PO_1/2	C/I?	57	GATCG|**gt**gtgaatttgccttctcca...ccttgctttggcgctcac**ag**|GTTCC
1	agil_WA	C	49	GATCG|**gt**gtgcatcgattcattctc...ctgacctccgaatgcgtc**ag**|GTTCC
3	agil_2970_1	C	44	TGGAG|**gt**ttgaaacacccgctgaaa...tgcccaaccctctctctc**ag**|CCTGG
3	agil_2970_2/3	C	47	TGGAG|**gt**ttgaaacacccgctggaa...ccaaccctctctcctctc**ag**|CCTGG
3	agil_323	C	46	TAGAG|**gt**acgctttgctggttgaaa...tctgtgtcgcctctcctc**ag**|CCCGG
3	agil_PR	C	50	TGGAG|**gt**atgctgctggctgactgt...tttcacttgcctcctctc**ag**|CCCGG
3	agil_WI	C	50	TGGAG|**gt**ttgccctccacctcactt...ctacaccttcgtttcctc**ag**|CCCGG
3	agil_PO_1	C	46	TGGAG|**gt**gtgctcagtttgttagtc...gctatctttcctcatttc**ag**|CCTGG
3	agil_PO_2	C	46	TGGAG|**gt**gtgctcagtttgttagtc...gttatctttcctcatttc**ag**|CCTGG
3	agil_WA	C	47	TGGAG|**gt**acgcctcagtcttgtgaa...tcacccgctttcactttc**ag**|CCTGG
9	agil_2970_1/2/3	I/N?	162	GCTCT|**gt**tctgagtgttgctgctgc...agcagcagcattcaggggtt|GACGG
9	agil_323	N	110	GCACT|atcctggccttttgctgcaa...acaacaaaaagccagtggtt|GACCG
9	agil_PR	N	91	GCACT|gacctggctctctgcctggg...ttgagcaggagccaggagta|GACAG
9	agil_WI	I/N?	542	GCTCT|**gc**cctggttgcctgctcatc...aggagcagtgaccagttgtg|GACGG
9	agil_PO_1/2	N	52	GCACT|gacctgactcgttgcttttc...tgaagcaacagccagtgttt|GACGG
9	agil_WA	I/N?	77	GCACT|**gt**cctggcttgttgctgaga...gacagagcaagccaggagtt|GACCG
15	agil_2970_1/2/3	N	305/304/304	GCAAC|agacaggctgacacaaaccg...cttcgtgtcgtcctgggagc|AACAC
15	agil_323	N	129	GCAAC|acacaggatggcaaagttgt...aaccctgccatactgagccc|AACAC
15	agil_PR	N	82	GCAAC|aagcaggatggcacggattg...atcagtgccgtcctgcacac|AACAC
15	agil_WI	N	224	GCAAC|aaacaggagagccaaggga...tcctctgttctcctgcacgt|AACAC
15	agil_PO_1/2	N	145	GCAAC|acacaggatggcactgtgcc...aaatgtgccatcctgcgccc|AATAC
15	agil_WA	N	132	GTAAC|aaccagggtgacaactgtgt...cggagtgtcaccctggagtc|AACAC

Supplementary Table S2. Exon-intron junctions in *hsp90* genes from six strains of *E. agilis*. For description see table S1.

hsp90

pos	name	type	size	sequence
3	agil_2970	N	54	GTGCC|gaacaggagccctcacagcc...gctgcaggtctcctgacttc|ATCCT
3	agil_323	N	55	GTGCC|tctcagaggtccctcaactt...gttgcaggacttctggctgc|ATCCT
3	agil_PR	N	53	GTGCT|cttcaggagtcacacagaca...actttctgacttctggccgc|ATCCT
3	agil_WI	N	56	GGGCT|aagcaggggagctctgcagt...ggcagagctctcctgcccgc|ATCCT
3	agil_PO	N	58	GTGCT|tctcaggagtctggcgtcgg...actgcaggactcctggcttc|ATCCT
3	agil_WA	N	59	GTGCT|tttcaggggtccagcaaaat...tttgctggacctctggctgc|ATCCT
5	agil_2970	N	1148	ACCGC|actcaggtttcattttcctt...tgcaaatcaaacctgcaaaa|AAGAA
5	agil_323	N	223	ACCGC|actcaggactcagcaaaatt...aaaaaatgagtcctgtccac|AAGAA
5	agil_PR	N	584	ACCGC|aaccaggactcagctgtttt...gtacaaggagccctgtccgc|AAGAA
5	agil_WI	N	737	ACCGC|acacaggtttcaggtcgagg...taaaaatgtgacctgcccgc|AAGAA
5	agil_PO	N	128	ACCGC|agccagggctcagtgtggtt...tccaaccgagccttgaaagc|AAGAA
5	agil_WA	N	125	ACCGC|agtcagggttcgcttaaggg...ccttgcagagctctgcacgc|AAGAA
6	agil_2970	I/N?	1077	GCGTG|**gc**ccaaaccttcacacagtg...ctttgtgaaagtttgtgtga|GATAT
6	agil_323	I/N?	256	GCGTG|**gc**acaagcatttgcagtcac...ggaggcaaagggttgcttgt|GATAT
6	agil_PR	I/N?	220	GCGTG|**gc**tcaagcttttgcacgtcc...aagtgcaaaagtttgccaat|GATAT
6	agil_WI	N	198	GCGGG|acccaaaccttgtctgattt...caatgccagggtttgtcagc|GATAT
6	agil_PO	I/N?	507	GCGTG|**gc**tcaggcctgcgcagcatg...ttgttgccaggcctgctcgt|GATAT
6	agil_WA	I/N?	153	GCGTG|**gc**tcaaacctttgcatttcc...ctgtgcaatggtttgcgggt|GATAT

7	agil_2970	N	384	AGACT|ggtcagtttccattcctcag...gcacagtggaatctgcagtc|GAGGA
7	agil_323	N	218	AGAGC|aacaaggtttcatttggtt...tgcaaatgtcacctgccgcc|GAAGA
7	agil_PR	I/N?	235	AGAGC|gccaaggttccattttactg...acagaagggaacctttcagc|GAGGA
7	agil_WI	N	258	AGACT|ggccagattccattgtttcc...cagaaatggagtctgtttga|GAGGA
7	agil_PO	N	182	AGACC|aactaggttcttgttctaac...gacgaacagaatctgtcgtc|GAGGA
7	agil_WA	N	140	AGAGC|agctaggttcttttctctca...ttgatatggaacctgccaac|GAGGA
8	agil_2970	N	1950	GCGAC|attcaggaggccattctggt...tgaaatgacctcctgatgtt|GCCTC
8	agil_323	I/N?	1953	GTGAT|gcgcaggaggtcatcttgt...ttggatggcctcctggtttt|GCCTC
8	agil_PR	N	1773	GTGAC|attcaggaggtcatccgtgg...tcggattgcctcctgcttct|GCCTC
8	agil_WI	N	438	GTGAC|agccaggaggccatctctct...taagatgacctcttgggttt|GCCTC
8	agil_PO	N	1575	GTGAC|aagcaggaggtcattaaaat...acatttgacctcctgttgtc|GCCTC
8	agil_WA	I/N?	1090	GTGAT|gcacaggtgctcattcaggt...ttggatgatcacctgcagtc|GCCTC
9	agil_2970	N	2343	CTGAC|aagcaggggtttttcacac...tgatgacgacccctgagacc|GAGGA
9	agil_323	C/I/N?	301	CAGAT|gcccagggctttcctcccgg...atgccaaggcccctgccgag|GAGGA
9	agil_PR	N	247	CTGAT|gagcaggggcctttcagtca...atccaaagggccctggattt|GAGGA
9	agil_WI	N	1128	GTGAC|agccaggaggccatctctct...tcagccaaccctctgtggtc|GAGGA
9	agil_PO	N	1894	GTGAC|aagcaggaggtcattaaaat...gtccaaaggcccctggagat|GAGGA
9	agil_WA	I/N?	1313	GTGAT|gcacaggtgctcattcaggt...cgcaaaaagcccctggcgct|GAGGA

Supplementary Table S3. Exon-intron junctions in *gapC* genes from six strains of *E. agilis*. For description see table S1.

				**gapC**
**pos**	**name**	**type**	**size**	**sequence**
1	agil_2970	C	52	ACCAG|gttgcactccaaaacgtgac...acctgttccatttccccag|ATGAA
1	agil_323_1/2	C	52	ATCAG|gtgtttttttggtgccaga...caaagttcaattttccgtag|ATGAA
1	agil_PR	C	52	ACCAG|gttcattgttgagttggaaa...tccccttgcattctttcag|ATGAA
1	agil_WI_1/2	C	47	ATCAG|gttcgtttgaaaacctttga...ctatgtccactttttcttag|ATGAA
1	agil_PO_1	C	50	ATCAG|gtgcgcttttcactggtaat...tggcacttctttttgtacag|ATGAA
1	agil_PO_2	C	48	ATCAG|gtgcgcttttcaccaggtgt...cactcacttcatactcacag|ATGAA
1	agil_WA	C	51	ACCAG|gtgcatcactgatctcgtct...tcattccatttgctttacag|ATGAA
2	agil_2970	N	52	ACCGC|aacaaaccctctgcttggca...acaagcagtgggttttcctt|GACCC
2	agil_323_1/2	N	71	ACCGC|agccaactccctgcttacag...acaagcagggagttttcctc|AACCC
2	agil_PR	N	80	ACTGC|aacaaaccccctgcttcttt...gtaagcattgggtttttctc|AACCC
2	agil_WI_1/2	N	56	ACCGC|accaagagcactgcttgaac...gcaagcagtggtctttccac|AACCC
2	agil_PO_1/2	N	64	ACTGC|aacaaactctctgcttggtg...acaagcagcgagttatttc|GACCC
2	agil_WA	N	68	ACTGC|actaagccctgcgcttgact...tcaatcactgggctttgtcc|AACTC
4	agil_323_1	N	162	GTCCC|aaccattaccttgtcctact...tgaagccaaggtatggtccc|AACTC
4	agil_323_2	N	160	GTCCC|aaccattaccttgtcctacc...tgaagccaaggtatggtccc|AACTC

Supplementary Table S4. Primers used for amplification and sequencing of *hsp90* and *gapC* genes.

PCR primers – *hsp90*	Sequence
EA9F0	ATGGCCGCWGAGACCTACACCTTCC
EA9R0	AAACCCCACAAACAGGCCAGAGTCAACC
EA9F1	ACACCTTCCAAGCTGAGATCAACCAGTT
EA9R1	AGAGAATTACTGTASCYGTGAAAGACTC
EA9sF0	GACCCGTGATCCCAAGGACATCAC
EA9sR0	CGGGGTCATCCAGGCTGAAGCC
EA9sF1	CTTCCTTCTACAAGGCCATCTCCAA
EA9sR1	CAGCAGCCACACCAAGTCTTTGAC
EA9sF2 (also sequencing primer)	GAGAACCTGTGCAAGCTGATCAA
EA9sR2 (also sequencing primer)	CATGATGCGCTCCATGTTGGC
EA9sF3	TGGGGGACAAGGTGGAGAAGGTGA
EA9sR3	GGTGACAAGGATGCAAGGGGAGTT
Universal internal sequencing primers – *hsp90*	
100XF	CAGCTGATGTCCCTGATCATYAAYACNTTYTA
975XF	MGCRCCTTTTGAYATGTTYGAGACMA
1297F	GAAGGARGGNCAGAAGGA
1297R	TNTCCTTCTGNCCYTCCTTC
950XR	TCCATBGTCTTCTTSGABAYCATGT
968XR	CCCAGCTTGATCATNCGRTGDAT
Specific internal sequencing primers – *hsp90*	
2970e3seqF (ACOI 2970)	CCACGAAGTCAGGCGAGGAGTC
2970i3seqF (ACOI 2970)	AATGTTACCCCTCCACCCAGTC
2970i3seqR (ACOI 2970)	TCACAAAGTTGCTCCAGAAG
2970i5seqF (ACOI 2970)	AGTGACTGGCTGGTTTTGATTA
2970i5seqR (ACOI 2970)	ACTGGGGCCTTGCGTCGTTGTT
2970e6seqF (ACOI 2970)	CTGATGTTGCCTCCACCTCCTCGTAT
2970i6seqF (ACOI 2970)	CAAGGGCTGGTCGCAAGAGG
2970i6seqR (ACOI 2970)	CAGGGGGTTGAAACAGGTCCAT
323i5seqF (ACOI 323)	AACCCAACCACCCACTTTCA
323i5seqR (ACOI 323)	GTTCGGCAAGGGGGGTTCGTT
WIi6seqF (WI)	GAACTCCCCGTGCATCCTGGTCA
WIi6seqR (WI)	ACTTGCGCCCCTCACCTGTTCATTAC
PCR primers – *gapC*	
EAgCF0	AGGCACTGTGCGACCAGGGACT
EAgCR0	TCTCGCCGGGCAAGTTGTTCTG
EAgCF1	TCGATATGGCCACTGATGCTGACT
EAgCR1	GTCGGTGGACACAAGCTCCTCATC
